# Estimating evolutionary and demographic parameters via ARG-derived IBD

**DOI:** 10.1371/journal.pgen.1011537

**Published:** 2025-01-08

**Authors:** Zhendong Huang, Jerome Kelleher, Yao-ban Chan, David Balding

**Affiliations:** 1 Melbourne Integrative Genomics, School of Mathematics & Statistics, University of Melbourne, Victoria, Australia; 2 Big Data Institute, Li Ka Shing Centre for Health Information and Discovery, University of Oxford, Oxford, United Kingdom; Case Western Reserve University, UNITED STATES OF AMERICA

## Abstract

Inference of evolutionary and demographic parameters from a sample of genome sequences often proceeds by first inferring identical-by-descent (IBD) genome segments. By exploiting efficient data encoding based on the ancestral recombination graph (ARG), we obtain three major advantages over current approaches: (i) no need to impose a length threshold on IBD segments, (ii) IBD can be defined without the hard-to-verify requirement of no recombination, and (iii) computation time can be reduced with little loss of statistical efficiency using only the IBD segments from a set of sequence pairs that scales linearly with sample size. We first demonstrate powerful inferences when true IBD information is available from simulated data. For IBD inferred from real data, we propose an approximate Bayesian computation inference algorithm and use it to show that even poorly-inferred short IBD segments can improve estimation. Our mutation-rate estimator achieves precision similar to a previously-published method despite a 4 000-fold reduction in data used for inference, and we identify significant differences between human populations. Computational cost limits model complexity in our approach, but we are able to incorporate unknown nuisance parameters and model misspecification, still finding improved parameter inference.

## Author summary

Samples of genome sequences can be informative about the history of the population from which they were drawn, and about mutation and other processes that led to the observed sequences. However, obtaining reliable inferences is challenging, because of the complexity of the underlying processes and the large amounts of sequence data that are often now available. A common approach to simplifying the data is to use only genome segments that are very similar between two sequences, called identical-by-descent (IBD). The longer the IBD segment the more informative it is about recent shared ancestry, and current approaches restrict attention to IBD segments above a length threshold. We instead are able to use IBD segments of any length, allowing us to extract much more information from the sequence data. To reduce the computational burden we identify subsets of the available sequence pairs that lead to little information loss. Our approach exploits recent advances in inferring the genealogical history underlying the sample of sequences. Computational cost still limits the size and complexity of problems our method can handle, but where feasible we obtain dramatic improvements in the power of inferences.

## Introduction

Multiple techniques have been developed for inference of evolutionary and demographic parameters such as the mutation rate or effective population sizes. Methods based on the sequential Markov coalescent (SMC) model [1–5] are typically likelihood-based, and hence statistically efficient but computationally demanding which restricts the sample sizes that can be handled. Other approaches can handle large sample sizes by using the allele frequency spectrum (AFS) [6–9], which reduces the genome sequences to counts of sites with each allele frequency. However, this data reduction loses statistical power, particularly for small sample sizes.

Another common approach to the analysis of genome sequence data is to first extract the lengths of genome segments that are inferred to be identical-by-descent (IBD) [10–14]. In practice, IBD is often identified by searching for regions with no evidence for recombination along two sequences since their most recent common ancestor (MRCA), which is problematic because recombinations can be hard to detect or even unobservable. Further, only IBD segments (IBDs) above a given length threshold, often 2 to 4 cM, are retained. This practice wastes valuable information, but has been necessary because the inference of short IBDs is too noisy to be useful for downstream analyses.

The ancestral recombination graph (ARG) is widely used to represent the genealogical history of a sample [15–17] and recent developments in inferring aspects of the ARG [18–22] now permit us to rapidly extract IBD directly from inferred shared ancestors [23], without requiring zero recombination. Further, ARG inference and IBD extraction are now fast enough to be implemented within an approximate Bayesian computation (ABC) algorithm [24]. Because ABC is simulation-based, requiring no knowledge of the likelihood function, even short inferred IBDs can contribute to inference, removing the need for an information-wasteful length threshold. Instead, we reduce computational cost by using an efficient subset of IBDs that scales linearly with sample size, resulting in little information loss relative to using all IBDs.

Our approach relies on a data structure encoding features of an ARG underlying a sample of genome sequences, called the succinct tree sequence (TS) [25, 26]. The TS minimises redundant storage of subsequences that are similar due to shared ancestry. It has led to spectacular improvements in storage and simulation of large genome datasets [27], and has recently been applied to IBD-based inferences about demographic history and evolutionary parameters [28].

**Fig 1:**
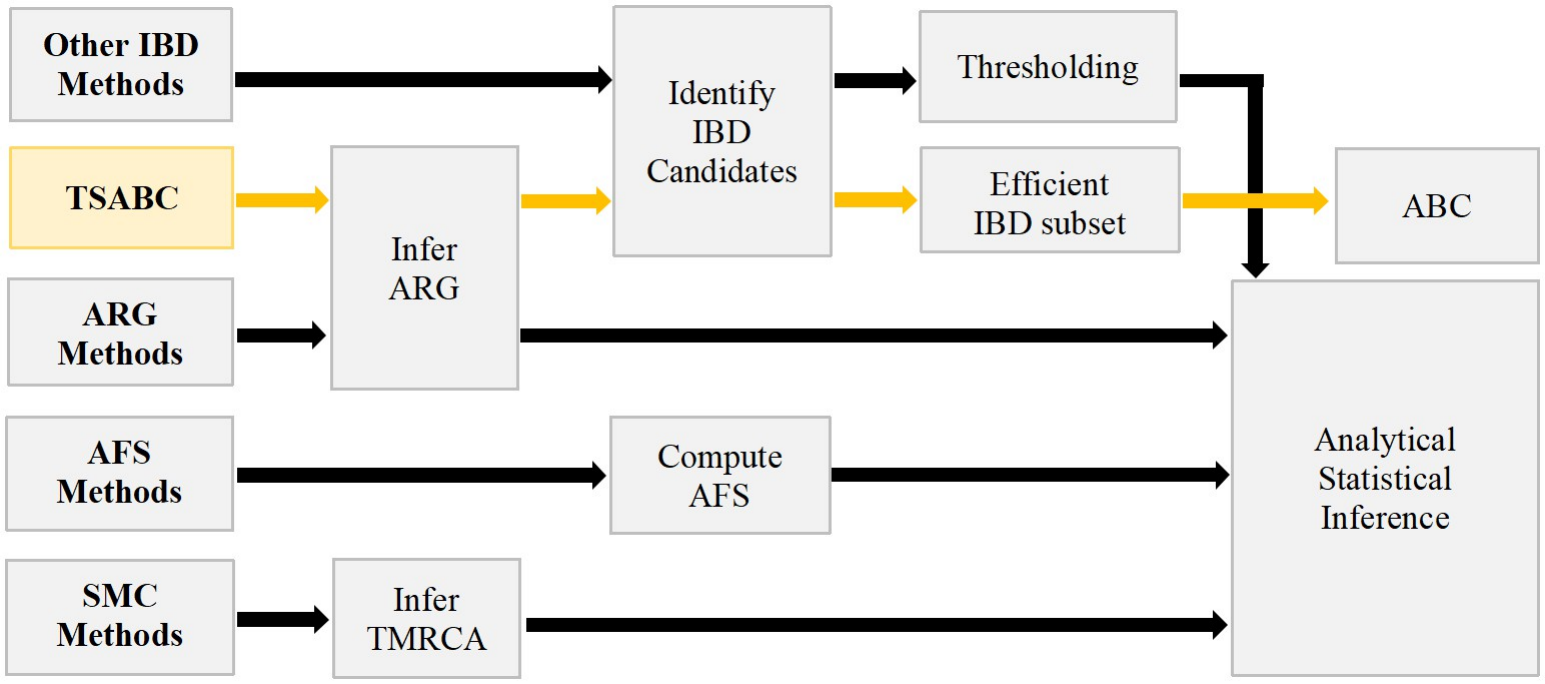
A sketch of different approaches to inference of evolutionary and demographic parameters from samples of genome sequences.

We first demonstrate powerful inferences of mutation and sequencing error rates, and past and present population sizes, given true IBD information in simulation studies. For real datasets, we propose TSABC: ABC with statistics computed from IBDs extracted from an inferred TS. Fig 1 summarises key features of TSABC and other methods. We demonstrate the performance of TSABC with inferences of the mutation rate and population size in simulation studies and real data, and we compare mutation rate estimates with previously-published results and with analyses using a range of IBD length thresholds.

We find that using IBDs extracted from an inferred ARG leads to a surprisingly small loss of precision in TSABC relative to use of true IBDs. Further, TSABC performs best with no IBD length threshold: even a low threshold on IBD length reduces the quality of inferences, despite the fact that short IBDs are poorly inferred. TSABC is computationally demanding, which limits the size and complexity of inference problems that can be tackled. However, TSABC can achieve comparable results to previous estimators while using much smaller data sets: we show similar precision to a previously-published mutation-rate estimator despite a 4 000 fold reduction in data available for inference (400-fold smaller sample size and 10-fold smaller genome length).

## Description of the method

### Definition and notations

The TS encodes genome sequence data efficiently by storing subsequences that are similar as variations of an inferred ancestral sequence. It is defined as {C,P,E,M}, where C={1,…,m} is the set of leaf (or tip) nodes corresponding to *m* observed sequences each of length *ℓ*, and *P* = {*m*+1, …, *n*} is the set of internal (ancestral) nodes of the TS ordered backwards in time from the present. The *j*th edge (*c*_*j*_, *p*_*j*_, *l*_*j*_, *r*_*j*_) in *E* represents inheritance of sites in the segment [*l*_*j*_, *r*_*j*_], with 1 ≤ *l*_*j*_ ≤ *r*_*j*_ ≤ *ℓ*, from internal node *p*_*j*_ ∈ *P* to its child *c*_*j*_ ∈ {1, …, *p*_*j*_−1}, while the *j*th element of M is a pair (*c*_*j*_, *s*_*j*_) recording the set of sites *s*_*j*_ at which there is a sequence difference between node *c*_*j*_ and its parent, due either to a mutation or, if *c*_*j*_ is a leaf node, sequencing error. The TS has the “succinct” property that any tree component conserved over a genome segment is stored only once, which greatly reduces data storage requirements compared with retaining all distinct marginal trees.

### Identity by descent and efficient subsets

We denote the *i*th IBD segment, *i* = 1, …, *I*, in the TS by IBD_*i*_ = (*c*_*i*1_, *c*_*i*2_, *l*_*i*_, *r*_*i*_, *p*_*i*_, *M*_*i*_), ordered such that *c*_*i*1_ is non-decreasing in *i*. Here *c*_*i*1_ and *c*_*i*2_ are the leaf nodes of the two sequences, [*l*_*i*_, *r*_*i*_] is the IBD genome segment, *p*_*i*_ is the MRCA node of *c*_*i*1_ and *c*_*i*2_ for this segment, and *M*_*i*_ denotes the set of sites in [*l*_*i*_, *r*_*i*_] at which *c*_*i*1_ and *c*_*i*2_ differ. We write *g*_*i*_ for the TMRCA (time since MRCA) of *c*_*i*1_ and *c*_*i*2_, which is the age of *p*_*i*_, in generations. As there is no length threshold, the IBDs of any sequence pair partition the genome: every sequence site is included in exactly one of the IBD segments.

**Fig 2:**
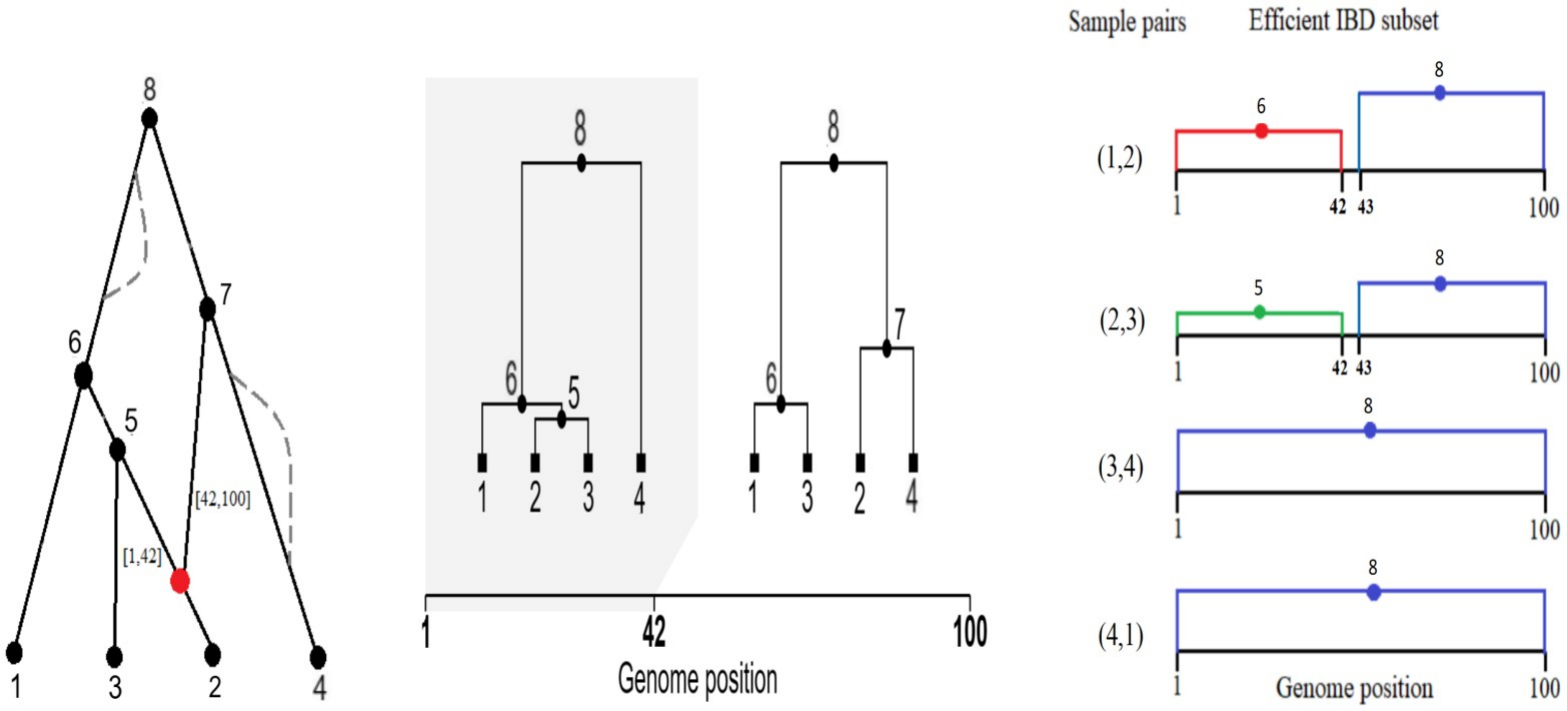
An ancestral recombination graph (ARG) spanning a genome sequence of length *ℓ* = 100 (left), the corresponding sequence of local trees (middle) and efficient IBD subset (right). The ARG has leaf nodes {1,2,3,4}=C, named ancestral nodes {5, 6, 7, 8} = *P*, and a recombination at site 42 (red node). The two dashed lines in the ARG represent inheritance paths due to two unobservable recombination events, which are not represented in the TS. The efficient IBD subset includes two IBD segments for the node pair (1, 2), corresponding to intervals [1, 42] and [43, 100] which have MRCA 6 and 8, respectively, and one IBD segment spanning the whole sequence for pairs (3, 4) and (4, 1). The sequence pairs (1,3) and (2,4) are not included in the efficient subset.

Each IBD_*i*_ has the same MRCA at each site in [*l*_*i*_, *r*_*i*_], and a different MRCA at adjacent sites. Imposing a no-recombination requirement as part of the definition of IBD would be more restrictive, since the absence of recombination implies a common MRCA but the reverse does not hold (see Fig 2, left, for examples of recombinations that do not change the MRCA).

To reduce computational effort, we use for inference only an “efficient” subset of IBDs. After fixing an arbitrary order for the sequences, we include in the subset only the IBDs of the sequence pairs (1, *m*) and (*c*, *c*+1) for *c* = 1, …, *m*−1 (see Fig 2, right, and S1 Text). An efficient subset has the property that each edge of the TS is included in a descent path from the MRCA for at least one IBD segment in the subset, which ensures that information is retained in the subset about every mutation.

Imposing a length threshold on IBDs is also a form of data reduction but we show below that it can lead to high information loss, because mutations are ignored if they occur at sites not contained in a sufficiently long IBD segment.

### Estimation

Let *μ* and *ϵ* be the per-site per-generation mutation rate and the per site sequencing error rate, both assumed constant over sites. Let *N*(*g*), *g* = 0, 1, 2, …, be the haploid population size *g* generations in the past. In S2 Text we derive method-of-moment estimators for *μ* and *ϵ*, and non-parametric estimators of *N*(*g*), *g* ≥ 0, based on statistics computed from IBDs. We investigate the performance of these estimators when true IBD information is available in simulation studies.

For observed sequence data, true IBD information is not available and we extract IBDs from an inferred TS. TSABC uses summary statistics derived from these IBDs and related to the method-of-moments estimators. In brief, the usual TSABC workflow is:

(i) from the observed *m* sequences, infer a TS;
(ii) for each of the *m* sequence pairs in the efficient subset, use the inferred TS to partition the sequence into segments with the same MRCA (IBD segments);
(iii) compute summary statistics from the IBD segments;
(iv) perform *η* times:
  (a) simulate a parameter value from a specified prior distribution,
  (b) use it to simulate a dataset of *m* sequences,
  (c) apply steps (i) through (iii) to the simulated dataset,
  (d) compute a distance *d* between the summary statistics of the observed dataset and those of the simulated dataset;
(v) the parameter values for the simulated datasets that have the smallest *d* values are retained as an approximate random sample from the posterior distribution.

See S1 Text for implementation details of the TSABC algorithm, including the linear adjustment [29] used to improve the posterior approximation in step (v). Unless otherwise stated, we used *η* = 2500 in step (iv) of which *η*/20 = 125 values are retained in step (v). Here we report only the mean of the retained parameter values, which estimates the posterior mean. Other properties of the posterior distribution can be approximated if desired.

We use tsinfer [19] in step (i); speed is critical for an ABC algorithm, and tsinfer is the fastest of the current methods, while retaining high accuracy [22, 30]. For inference of *μ* and *ϵ*, we use the statistics $\bar{M}\times I$ and *C*_1_ (S2 Text) which are constructed from the method-of-moments estimators $\hat{\mu }$ and $\hat{\epsilon }$. Nonparametric estimation of *N*(*g*) is not feasible, but we can estimate the parameters of a demographic model, which allows powerful inference provided that the model is adequate. We use as statistics in the ABC algorithm the mean and standard deviation (SD) of IBD lengths *r*_*i*_ − *l*_*i*_, *i* = 1, …, *I*.

The recombination rate *r* is assumed constant over sites and known for analyses of simulated data, whereas a previously-published human recombination map is assumed for the real-data analysis. A recombination at site *s* means between sites *s* and *s* + 1.

## Verification and comparison

### Simulation study: True IBD available

We used msprime [32] to simulate TS under the coalescent with recombination [33, 34], assuming demographic models C, Ga and S (Table 1). Sequencing error was simulated by adding elements to M at the TS leaf nodes. At the largest error rate (*ϵ* = 10^−3^), when *μ* = 1.3×10^−8^ any singleton variant is a few times more likely to arise from sequencing error rather than a mutation. For each simulated dataset we estimated *μ*, *ϵ*, and *N*(*g*), *g* ≥ 0, using our novel estimators (S2 Text).

**Table 1:** Parameter values, sample properties and demographic models for the simulation study. Unless otherwise stated, 25 simulation replicates were generated in each scenario. Model Ga is used for inferences given true IBD and Model Gb is used for inferences from inferred IBD. The value of *r* is assumed known for all inferences, whereas *μ*, *ϵ* and *N*(*g*), *g* ≥ 0, are targets of inference.

Evolutionary parameters and sample properties		
Symbol	Definition	Value(s) in simulations
*μ*	mutation rate	1.3×10^−8^ per site per generation (fourfold lower and higher in Table 3)
*ϵ*	sequencing error rate	up to 10^−3^ per site
*r*	recombination rate	10^−8^ per site per generation
*m*	sample size	from 10 to 200 sequences
*ℓ*	sequence length	from 10^6^ to 10^8^ sites
Demographic models (*N*(*g*) = haploid population size *g* generations ago)		
Model C	*N*(*g*) constant	*N*(*g*) = 2×10^4^
Model G	*N*(*g*) = *N*(0) × *e*^−*τg*^	*N*(0) = 10^6^, *τ* = 10^−4^ (Model Ga) *N*(0) = 2×10^5^, *τ* = 10^−3^ (Model Gb)
Model S	*N*(*g*) = *N*(0) for 0 < *g* < g* = *N*(g*) for *g* ≥ g*	*N*(0) = g* = 4×10^4^ *N*(g*) = 10^4^
Model EA	European-American demographic history [31]	See S1 Fig for *N*(*g*) values; gene conversion: tract length 300 bp rate 2×10^−8^/site/generation.

**Fig 3:**
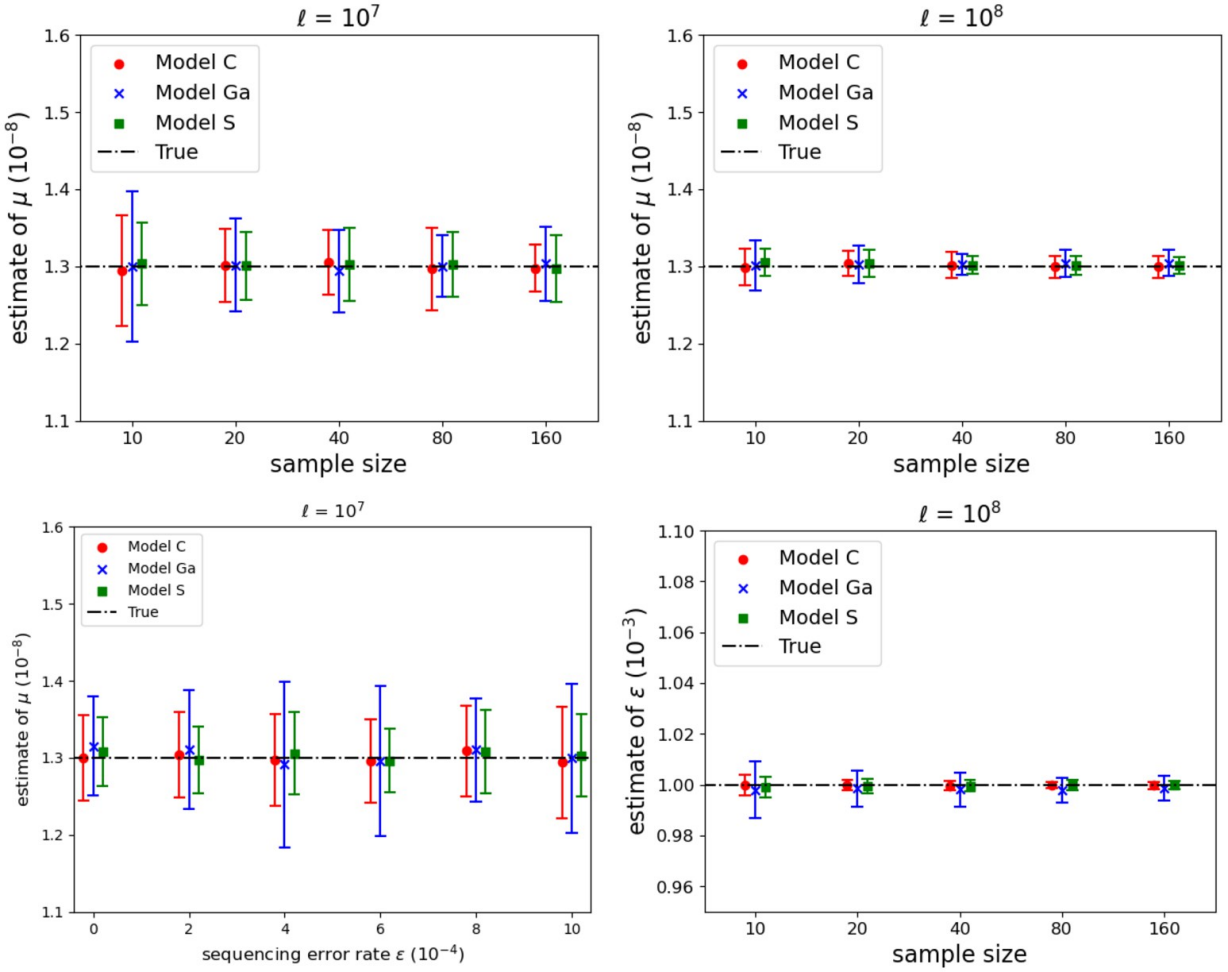
Inference of mutation rate *μ* and sequencing error rate *ϵ* with two sequence lengths (columns), when true IBD was available for inference. Line segments show indicative 95% CIs computed from the average estimate (indicated by a symbol, see legend box) and the empirical SD of the estimates from 25 simulated datasets in each scenario. Bottom left panel shows the impact of *ϵ* on $\hat{\mu }$ when *m* = 10, see S2 Fig (right) for corresponding results when *ℓ* = 10^8^. For the other three panels *ϵ* = 10^−3^.

Both $\hat{\mu }$ and $\hat{\epsilon }$ are well estimated in all demographic models, with no indication of bias (Fig 3). Increasing *m* has only a modest effect on the SD of estimators, whereas *ℓ* has a larger effect (SD scales with $\sqrt{\ell }$). Sequencing errors only inflate the number of singleton variants, so $\hat{\mu }$ is little affected by increasing *ϵ* (Fig 3, bottom left).

While use of the efficient subset of IBDs reduces computational cost in proportion to the reduction in sequence pairs from *m*(*m*−1)/2 to *m*, the average estimated SD of $\hat{\mu }$ in our Model C simulations increased only slightly, from 0.017 to 0.019 units of 10^−8^ (see S2 Fig, left, for confidence intervals (CI)). This gain in computation time is typically worth the small loss of statistical efficiency.

**Fig 4:**
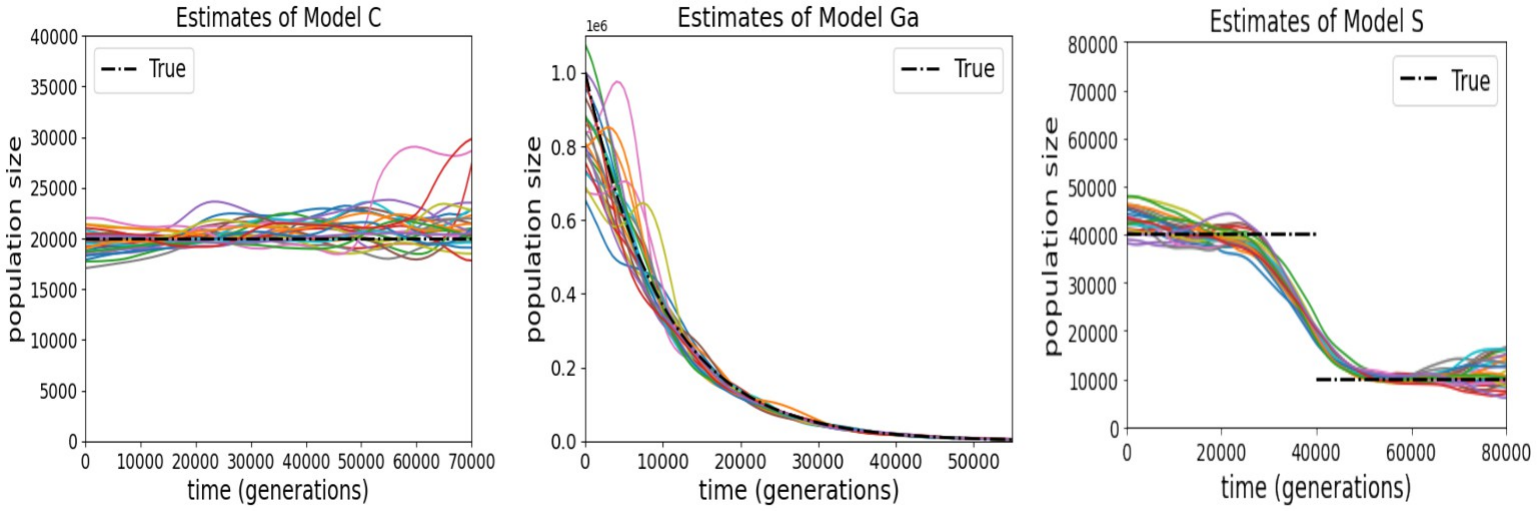
Estimates of the population size *N*(*g*), *g* ≥ 0, from each of 25 simulation replicates under Model C, Model Ga and Model S, when true IBD was available for inference. Sequence length is *ℓ* = 10^8^, sequencing error rate is *ϵ* = 10^−3^ and sample size is *m* = 80. See S3 Fig for corresponding results when *ℓ* = 10^7^.

The population size estimator $\hat{N}\left(g\right)$ is accurate under all models, at least for *g* ≤ 5 × 10^4^ (Fig 4). These inferences depend on the empirical densities derived from the ${\hat{g}}_{i}$, *i* = 1…, *I*, which are close to the theoretical densities (S4 Fig) despite imprecision of the individual ${\hat{g}}_{i}$ and the input TS only including information about the order of the coalescence events, and not their times.

### Simulation study: Inferred IBD

We used msprime to simulate sequences, recoded them as binary strings with 0 denoting the ancestral allele, and added sequencing errors by assigning 1 to randomly selected sites at rate *ϵ* (see S3 Text and [35] for alternative models of sequencing error).

We first use simulations to confirm a previous report [36] that the quality of IBD inference is often poor, particularly for short IBDs. We compared the number of true and inferred IBDs for datasets simulated under Model C with *μ* ranging from 1 to 20 units of 10^−8^ per site per generation and *m* = 10, 20 and 160. We also compared the length distribution of true and inferred IBDs for *m* = 160 and *μ* = 1.3×10^−8^.

**Fig 5:**
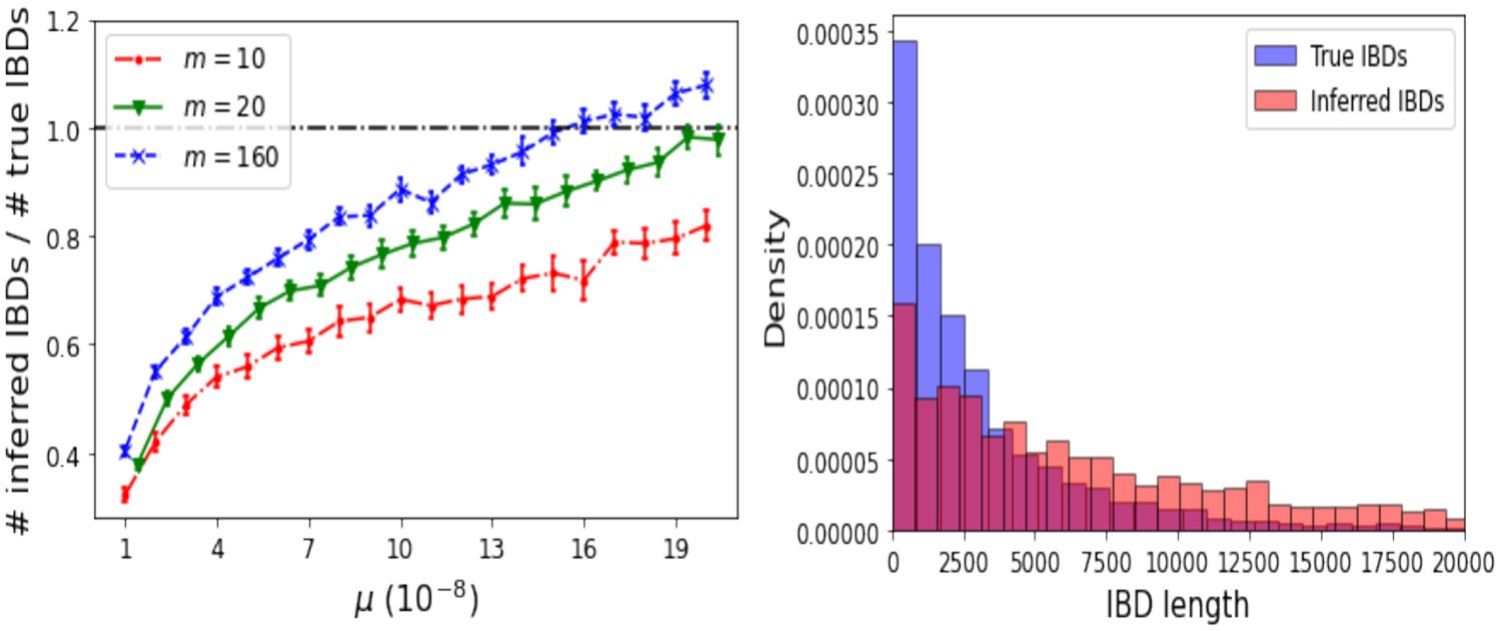
Comparison of true and inferred IBDs. Left: each symbol and vertical line segment show the mean and 95% CI of the mean ratio of IBD counts over 25 Model C simulations with sample sizes *m* = 10, 20 and 160. The human mutation rate is close to the left endpoints of the curves. Right: histograms of true and inferred IBD length distributions for a Model C simulated dataset with *m* = 160 and sequence length *ℓ* = 10^6^.

The number of inferred IBDs tends to increase with both *μ* and *m*, but except for very high *μ* (over 10 times the average human value when *m* = 160) it remains well below the true number of IBDs (Fig 5, left). Correspondingly, the length distribution of inferred IBDs is highly skewed towards larger values relative to the true distribution (Fig 5, right), as previously reported [36].

This poor inference of IBDs has motivated the widespread use of a length threshold to exclude short IBDs. We investigated its effect for the Model C simulations with *m* = 10 and *μ* = 1.3×10^−8^, modifying TSABC to include only IBDs longer than a threshold of 1, 2 or 4 units of 10^4^ bp. These thresholds are two orders of magnitude shorter than those typically used in practice so our results are likely to greatly understate any actual information loss from thresholding. When a threshold was applied, we included all IBDs satisfying the threshold, rather than using only the efficient subset of IBDs. For comparison, we also applied TSABC to estimate *μ* using IBD extracted from the true TS generated in simulations. Here, the TSABC workflow uses true rather than inferred IBD in step (ii) above, and hence also in (c), so that the data simulation within ABC mimics the generation process for the data treated as observed.

Table 2 shows that each decrease in the length threshold of IBDs used for inference of *μ* increased the resulting precision, both for inferred and true IBD, so that even poorly-inferred short IBDs improve TSABC inference. We also see in Table 2 (final column) further evidence that use of the efficient subset of IBDs leads to only a small loss of statistical efficiency. As expected, the use of true IBD improves TSABC compared with using inferred IBD, but the magnitude of the improvement is modest for low or zero threshold. For higher thresholds, bias can be high due to low precision of inference and the prior boundary at 10^−8^.

**Table 2:** Comparison of TSABC inference for *μ* using different IBD length thresholds. Each result is an average over 25 Model C simulation replicates with *m* = 10, *ϵ* = 0, *ℓ* = 10^7^ and *μ* = 1.3×10^−8^. In the last column, values based only on IBDs in the efficient subset are given in (). See S5 Fig for a plot of CIs.

Threshold (10^4^ bp)	4	2	1	0	
inferred IBD					
# IBD	1 001	7 033	24 683	48 289	(10 667)
$\hat{\mu }$ (10^−8^)	1.51	1.44	1.32	1.30	(1.31)
SD (10^−8^)	0.219	0.167	0.067	0.041	(0.043)
true IBD					
# IBD	478	1 803	6 940	121 027	(26 394)
$\hat{\mu }$ (10^−8^)	1.33	1.30	1.31	1.30	(1.29)
SD (10^−8^)	0.141	0.089	0.064	0.029	(0.034)

We next investigated TSABC estimation of *μ* under Model C and Model Gb with *μ* set to 0.25, 1 and 4 times the human value of 1.3×10^−8^, approximately spanning the range of *μ* in vertebrates [37], and *ℓ* set to 4, 1 and 0.25 times 10^7^. The expected number of mutations is the same in all scenarios but when *μ* is higher more of the mutations arise as multiple hits at the same site, while lower *ℓ* means fewer recombinations. The *N*(*g*) values and *ϵ* = 0 were assumed known, and the prior distribution for *μ* when the true value was 1.3×10^−8^ was Uniform(10^−8^, 2×10^−8^), with the endpoints of the uniform prior changing in proportion when *μ* was fourfold lower or fourfold higher.

Bias in TSABC estimation appears to be negligible for all *μ* values (Table 3). The loss of precision (increased SD) as *μ* increases and *ℓ* decreases is due both to fewer recombinations, which reduces precision through less independence along the genome, and more sites with multiple mutations, which are less informative for inference than if the mutations had occurred at distinct sites.

**Table 3:** TSABC estimation of mutation rate *μ*. The expected number of mutations is the same in each scenario (*μ* × *ℓ* is constant). Values are averages over 25 simulations with no sequencing error (*ϵ* = 0).

Sample size	10	20	40	80
*μ* = 0.325 × 10^−8^, *ℓ* = 4 × 10^7^				
Model C	0.321 (0.007)	0.326 (0.005)	0.325 (0.005)	0.326 (0.005)
Model Gb	0.325 (0.005)	0.326 (0.005)	0.327 (0.005)	0.326 (0.004)
*μ* = 1.3 × 10^−8^, *ℓ* = 10^7^				
Model C	1.312 (0.032)	1.304 (0.032)	1.304 (0.025)	1.301 (0.027)
Model Gb	1.293 (0.027)	1.306 (0.016)	1.298 (0.016)	1.299 (0.021)
*μ* = 5.2 × 10^−8^, *ℓ* = 0.25 × 10^7^				
Model C	5.320 (0.317)	5.211 (0.250)	5.209 (0.219)	5.239 (0.195)
Model Gb	5.163 (0.154)	5.193 (0.109)	5.219 (0.082)	5.215 (0.126)

To study TSABC estimation of the population size *N*(*g*), we used *m* = 200 and *ℓ* = 10^6^ under each of Model C and Model Gb. For both data simulation models, the TSABC inference used Model G but with different prior distributions. When the simulation used Model C, we fitted Model G with independent priors Uniform(10^4^, 3 × 10^4^) for *N*(0) and Uniform(−2×10^−5^, 2×10^−5^) for *τ*. Whenever *τ* < 0, we impose a population size limit *N*(*g*) ≤ 2*N*(0). When the simulation used Model Gb, the independent priors were Uniform(10^5^, 3×10^5^) for *N*(0), and Uniform(0, 0.002) for *τ*. All parameters were treated as known except the targets of inference *N*(0) and *τ*.

**Fig 6:**
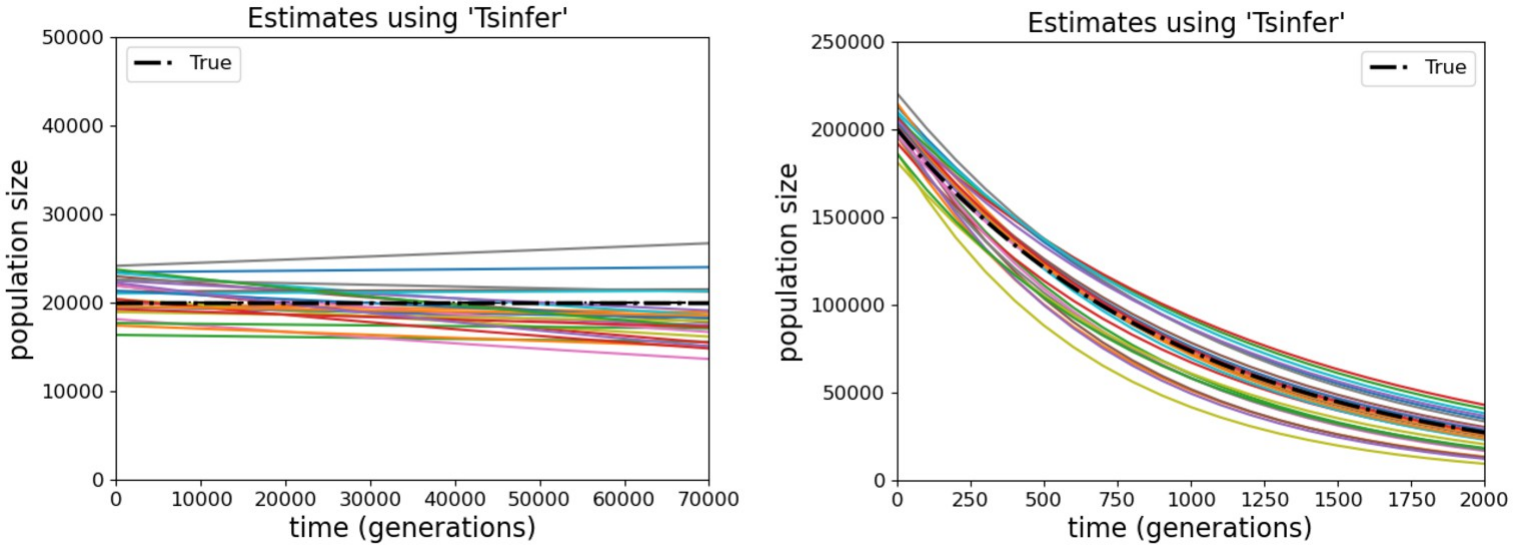
Fitted exponential curves for the population size *N*(*g*) obtained using TSABC. Each of the 25 curves corresponds to a dataset simulated under Model C (left) and Model Gb (right) with no sequencing error (*ϵ* = 0), sample size *m* = 200 and sequence length *ℓ* = 10^6^.

Results for parametric estimation of *N*(*g*) are shown in Fig 6. For Model C data simulations, the average estimate of *N*(0) (true value 20 000) over the 25 replicates is 20 931 with standard error (SE) $1\;428/\sqrt{25}=286$, while for the growth rate *τ* (true value 0) the average estimate is 2.10 with SE $1.8/\sqrt{25}=0.36$, both in units of 10^−6^. With Model Gb data simulations, for *N*(0) (true value 200 000) we obtained 202 534 (SE 2173) while for *τ* (true value 1) we obtained 1.08 (SE 0.07) in units of 10^−3^.

We performed additional simulations to allow the inferences of *μ* using the 3-way IBD method [31, 38] to be compared with Relate [20] and TSABC. Genomes consisting of 30 chromosomes were simulated under Model EA, which aims to capture key features of the demographic history of European-Americans (Table 1), and Model C.

The Model C simulations of [38] used *ϵ* = 10^−4^ but no gene conversion, while for Model EA [31] also included gene conversion with rate 2×10^−8^ per base pair per generation and mean tract length 300 bp. The data sets simulated for TSABC used the same sequencing error and gene conversion settings as [38] for Model C and [31] for Model EA. However TSABC was challenged by not including gene conversion in the inference model and by treating both *ϵ* and *N*(*g*) as unknown when inferring *μ*, with a misspecified model for *N*(*g*). The simulated datasets analysed by TSABC and Relate had a 400-fold smaller sample size than [38] (*m* = 10 versus *m* = 4×10^3^) and 10-fold smaller genome length (*ℓ* = 10^7^ per chromosome, versus *ℓ* = 10^8^).

When the data were simulated under Model C, TSABC used independent priors Uniform(10^−8^, 2×10^−8^) for *μ* and Uniform(0.6×10^−4^, 1.6×10^−4^) for *ϵ*. For *N*(*g*), we adopted Model G with independent priors *N*(0) ∼ Uniform(1.4×10^4^, 3×10^4^) and *τ* ∼ Uniform(−2×10^−5^, 10^−5^). When the data were simulated under Model EA, TSABC used the same priors for *μ* and *ϵ* as in Model C. For inference of *N*(*g*), we adopted Model S with independent priors *N*(0) ∼ Uniform(1.1×10^4^, 1.5×10^4^), g*∼ Uniform(4500, 6500) and *N*(g*)∼ Uniform(4.6×10^4^, 5.0×10^4^).

For Relate, we only report results from datasets simulated with neither sequencing error nor gene conversion, because it performed poorly on datasets with these features. We re-formatted the simulated sequences as .vcf files for input to Relate. The software RelateMutationRate (see https://myersgroup.github.io/relate) was then implemented to find the average mutation rates, with mode parameter “AVG”, bins parameter (4, 7, 10^7^), true values used for other evolutionary parameters, and default settings for other tuning parameters.

Table 4 shows that TSABC performs similarly to the 3-way IBD results reported by [31, 38] despite a 400-fold smaller sample size and a 10-fold reduction in sequence length, and despite the challenges we imposed on TSABC: sequencing error was treated as unknown and gene conversion was incorporated in data simulation but not the ABC inference model, which also misspecified the model for *N*(*g*). TSABC also provides more accurate inference for *μ* than Relate when analysing the same data, despite gene conversions and sequencing errors challenging TSABC but not Relate.

**Table 4:** Comparison of inference of *μ* (in units of 10^−8^, true value 1.3). 3-way IBD results are from [38] for Model C and [31] for Model EA. Relate and TSABC results are obtained from 25 simulated datasets under each model, with genomes consisting of 30 chromosomes each of length *ℓ*. The TSABC simulations included sequencing error and gene conversion with the same settings as [38] for Model C and [31] for Model EA. Relate performed poorly on those datasets and the reported results are for datasets simulated without sequencing error or gene conversion. For TSABC, $\hat{\epsilon }=1.03$ (SE 0.03) units of 10^−4^ for Model C, and 1.03 (SE 0.03) for Model EA (true value 1).

	Model C		Model EA		*m*	*ℓ*
	$\hat{\mu }$	SD	$\hat{\mu }$	SD		
3-way IBD	1.30	0.020	1.31	0.013	4 000	10^8^
Relate	1.31	0.023	1.26	0.024	10	10^7^
TSABC	1.30	0.009	1.31	0.017	10	10^7^

### Application: Mutation and growth rates in the 1000 Genomes Project

We analyse chromosomes 20 and 21 from 1 538 individuals in eight of the 26 human populations of the 1000 Genomes Project (1KGP) [39]. S4 Text gives details of the data analysis. Separately for each chromosome, we use TSABC to infer *μ* assuming the prior Uniform(10^−8^, 2×10^−8^), *ϵ* = 0 and the demographic model of [40], which we refer to as the 1KGP model (see S1 Fig for plots). The 16 sets of 125 retained values were analysed in a two-way ANOVA to assess differences in *μ* across chromosomes and over populations.

The global mean $\hat{\mu }$ over both chromosomes is 1.27 (SE 0.03) units of 10^−8^ per site and per generation, which is close to the whole-genome estimate 1.24 (SE 0.04) based on three samples, of European (2) and African (1) ancestries, totalling about 8.7K individuals [31]. These authors reported no significant difference across their three populations whereas our two-way ANOVA (Table 5) revealed highly significant differences, with population-specific estimates ranging from 1.22 (BEB, CHB) to 1.36 (GBR). These differences may be due to differences in heritable factors, average age at reproduction [41] or environmental exposures such as mutagenic solar radiation [42]. These population-based estimates are higher than current pedigree-based estimates, which are typically between 1.1 and 1.2 units of 10^−8^ [43, 44], but lower than estimates based on inter-species comparisons [45], consistent with a decrease in *μ* over time. We found no significant difference overall between the two chromosomes, but there is a highly-significant between-chromosome difference in ITU.

**Table 5:** Estimation of the mutation rate *μ* per site per generation (in units of 10^−8^) on human chromosome 20 and 21 for populations MSL (Mende in Sierra Leone), LWK (Luhya in Webuye, Kenya), BEB (Bengali from Bangladesh), ITU (Indian Telugu from the UK), FIN (Finnish in Finland), GBR (British in England and Scotland), JPT (Japanese in Tokyo, Japan), and CHB (Han Chinese in Beijing, China). The TSABC analysis assumes the 1KGP demographic model in each population.

		MSL	LWK	BEB	ITU	FIN	GBR	JPT	CHB
Sample size (*m*)		170	198	172	204	198	182	208	206
Chr 20	$\hat{\mu }$	1.27	1.24	1.23	1.22	1.32	1.36	1.32	1.20
	SE	0.004	0.004	0.004	0.004	0.004	0.011	0.004	0.004
Chr 21	$\hat{\mu }$	1.25	1.26	1.21	1.29	1.29	1.35	1.33	1.24
	SE	0.004	0.005	0.006	0.005	0.006	0.006	0.006	0.006
Combined	$\hat{\mu }$	1.26	1.25	1.22	1.25	1.31	1.36	1.33	1.22
	SE	0.003	0.003	0.003	0.004	0.004	0.006	0.004	0.004

To estimate population size *N*(*g*), 0 ≤ *g* ≤ 1000 (approximately 27 000 years [46]), we assume demographic Model G constrained such that *N*(1000) matches the 1KGP model value. The constrained Model G has one free parameter *N*(0), for which we adopt a Uniform(10^4^, 24×10^4^) prior. To reduce computational effort with little loss of information, in both the observed dataset and TSABC simulations we removed SNPs with minor allele count > 40, which typically correspond to mutations at *g* ≫ 1000. We estimate *N*(*g*) from each chromosome separately and average the results.

**Fig 7:**
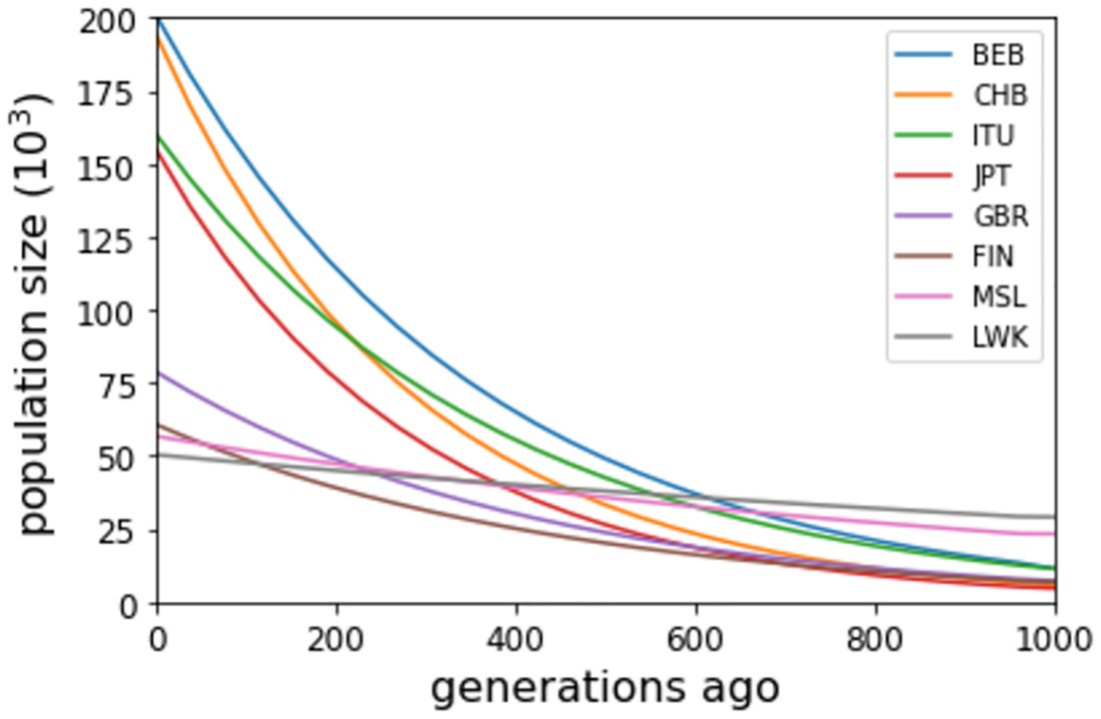
Estimates of recent population sizes for eight populations sampled in the 1000 Genomes Project. In the legend box, populations are listed in order of decreasing *N*(0). See Table 5 caption for explanation of the population labels.

Fig 7 shows positive growth in the past 1000 generations for all eight populations. CHB and BEB have the highest *N*(0), while MSL and LWK have the lowest *N*(0) despite having the highest values of *N*(1000). Previous studies have reported similar rapid growth in non-African populations for 0 ≤ *g* ≤ 1000 [2, 5, 20]. Estimates of *N*(*g*) can be sensitive to modelling assumptions and vary widely over studies, particularly for *g* < 400. However, ordering across populations is more stable. Our *N*(200) estimates are in the same order as [2] for the three populations (CHB, JPT, LWK) investigated in both papers, and in the same order as [20] for the four populations in common (CHB, JPT, FIN, GBR).

## Discussion

We have shown that ARG-derived IBD combined with ABC can deliver advantages over previous IBD-based methods for inferring mutation rate and population sizes from a sample of genome-wide sequences. Despite verifying that IBD extracted from an inferred TS is often inaccurate, TSABC showed only a modest loss of efficiency relative to the corresponding inference based on true IBD. Note again that the data simulation within ABC mimics the data generation process, and so differs between inferred and true IBD. Simply inserting the true IBDs into TSABC which assumes inferred IBDs would be inappropriate and generate poor results.

For simulated data, we found similar precision in estimating *μ* to previous results [31, 38] that used 4 000 times more data for inference. Similarly for real human data, we report better precision than [31] despite a five-fold smaller sample size and only chromosomes 21 and 22 rather than the whole genome. Further, we showed highly-significant differences in *μ* across human populations, but no overall significant difference across chromosomes, and our inferences of historic population sizes showed concordance with previous studies.

These advantages arise because from an ARG we can extract IBD defined in terms of a common MRCA, avoiding the problem of detecting recombinations, and we require only IBDs from *m* sequence pairs, rather than all *m*(*m*−1)/2 pairs, which reduces computational effort with little loss of statistical efficiency. The efficiency of ARG-based IBD inference is a key factor, allowing analyses of many simulated datasets, as required for ABC. Perhaps the most important advantage of TSABC derives from dispensing with the need for an IBD length threshold, allowing information to be extracted from the large number of short IBDs even though they are poorly inferred. A key to understanding why this is possible is that ABC inference does not require the summary statistics to be an accurate estimator of anything, nor do we need to know any distributional properties, we just need the distribution of the summary statistics to be sensitive to the target parameters.

Limitations of TSABC include those that apply to any ABC algorithm: the results are approximate and it can be difficult to estimate the approximation error. Another source of error comes from use of tsinfer for TS inference: more accurate inference is possible but alternative methods lack the computational efficiency of tsinfer. TSABC can be computationally demanding for complex demographic models, and the results presented here are limited to inferring one or two parameters. Although joint estimation of multiple parameters will be computationally challenging, they can be estimated iteratively, fixing some parameters while estimating others. TSABC is able to handle much larger datasets, both in terms of sample size and sequence length, than our previous likelihood-based approach [21]. Further, we were able to incorporate unknown nuisance parameters such as the sequence error rate and misspecification of the demographic model to challenge TSABC inference without substantial detriment to inference quality.

In addition to ABC-based inference, we also derived method-of-moment estimators for ARG-derived IBD, and showed in a simulation study based on true IBD that these estimators could provide very powerful inferences given an accurately inferred ARG. As ARG inference methods improve in accuracy, they may become directly useful on real data, but here we used them indirectly to develop the TSABC summary statistics.

Overall, our results open the way for more powerful evolutionary and demographic inferences from samples of genome sequences than have previously been available. Summary statistics based on IBD lengths represent one approach to extracting information from an ARG inferred from observed sequences, future developments may be based on better summaries, or directly on the ARG which presents challenges due to its complexity.

## Supporting information

S1 Text The efficient IBD subsets and ABC algorithms. (PDF)

S2 Text Derivation of estimators when TS is known. (PDF)

S3 Text Different models for sequencing error. (PDF)

S4 Text Further details for 1KGP data analysis. (PDF)

S1 Fig The 1KGP *N*(*g*) models. Natural logarithms of *g* are shown on the *x*-axis, with the models starting at *g* = exp(6) ≈ 400 generations in the past. The values of *N*(1000) which form the right endpoints of Fig 7 correspond to *x* = log(1000) ≈ 6.9.
(TIF)

S2 FigLeft: Estimated 95% CIs for the estimation of *μ* when an efficient subset of IBD segments was extracted from the TS and when all IBDs were used. At each sample size, 25 replicate datasets were simulated under Model C, with sequence length *ℓ* = 10^7^. Right: Impact of sequencing error rate *ϵ* on $\hat{\mu }$ when *ℓ* = 10^8^ (other details are the same as for bottom left panel of Fig 3).(TIF)

S3 Fig Estimates of the population size *N*(*g*) when *ℓ* = 10^7^ (other details are the same as for Fig 4). (TIF)

S4 FigHistogram of the $\hat{g_{i}}$, *i* = 1, …, *I*, obtained from one sample simulated under each of Model C (left) and Model Ga (right), with sample size *m* = 80, sequence length *ℓ* = 10^8^ and sequencing error rate *ϵ* = 10^−3^.Also shown is a probability density obtained by kernel smoothing of the $\hat{g_{i}}$ together with the true density. True IBD was available for inference but no time information.(TIF)

S5 Fig95% CIs computed from the estimates $\hat{\mu }$ and SE shown in Table 2.(TIF)
