# Supplementary material for: Estimating evolutionary and demographic parameters via ARG-derived IBD
Source: PLoS Genet. 2025 Jan 8;21(1):e1011537. doi: 10.1371/journal.pgen.1011537 (PMC11750106; doi:10.1371/journal.pgen.1011537)
Supplement: S1 Text — (PDF) [file pgen.1011537.s001.pdf]

Estimating evolutionary and demographic parameters via ARG-derived IBD

Text S1: Algorithms to extract IBD segments and perform ABC

The following algorithm is usually applied to all sequence pairs in the efficient subset.

Algorithm 1 Extracting IBD segments of sequences c_1 and c_2

1: Initialize the set of IBDs: $S = \emptyset$. Initialize the index of the first IBD: $i = 1$, the left endpoint of the first IBD: $l_1 = 1$, the current sequence site: $s = 1$. Find p_1, the MRCA of c_1 and c_2 at site 1.
2: Find the next site $s' > s$ such that the marginal tree of the sample set $\mathcal{C}$ remains the same at all sites between s and $s'-1$, but changes at s'. Find $p_{s'}$, the MRCA of c_1 and c_2 at site s'.
3: If $s' \leq \ell$ and $p_s = p_{s'}$, let $s := s'$ and go to Step 2.
4: If $s' > \ell$ or $p_{s'} \neq p_s$, let $r_i = s'-1$. Compute M_i, the number of site differences between c_1 and c_2 in (l_i, r_i). Add $\text{IBD}_i = (c_1, c_2, l_i, r_i, p_s, M_i)$ to set S.
5: Let $i := i+1$, $s := s'$ and $l_i = s$. If $s \leq \ell$, go to Step 2, else return the IBD set S and stop.

Within TSABC is a standard ABC algorithm, the innovation here is in the choice of summary statistics which are derived from IBD lengths extracted from an inferred TS.

Algorithm 2 ABC algorithm for parameter θ

1: Initialization. Set the number of simulation replicates η, and a prior distribution for θ with density f_θ. Write s_0 for the summary statistics computed from the observed data. Set a tolerance level $\varepsilon > 0$.
2: For $i = 1, \ldots, \eta$, generate θ_i from f_θ. With $\theta = \theta_i$ simulate a sample of m genomes of length ℓ, infer the TS from the simulated genomes, extract IBD segments in the efficient subset, and compute the summary statistics s_i. Apply the linear adjustment to θ_i by letting $\theta_i^* = \theta_i - (s_i - s_0)'\hat{\beta}$, where $(\hat{\alpha}, \hat{\beta}')' = \text{argmin}_{(\alpha,\beta)} \sum_{i=1}^{\eta} \{\theta_i - \alpha - (s_i - s_0)'\beta\}^2$.
3: Retain θ_i^* if $d(s_i, s_0) < \varepsilon$, where $d(\cdot)$ is the Mahalanobis distance.
