# Supplementary material for: Estimating evolutionary and demographic parameters via ARG-derived IBD
Source: PLoS Genet. 2025 Jan 8;21(1):e1011537. doi: 10.1371/journal.pgen.1011537 (PMC11750106; doi:10.1371/journal.pgen.1011537)
Supplement: S2 Text — (PDF) [file pgen.1011537.s002.pdf]

Estimating evolutionary and demographic parameters via ARG-derived IBD

Text S2: Derivation of Estimators when TS is known

Mutation and sequencing error rates

The right endpoint r_i of an IBD segment is usually the site of an effective recombination event for (c_1, c_2), where "effective" means that c_1 and c_2 have a different MRCA on either side. The exceptions are IBDs terminating at the sequence end site ℓ, which are excluded from the derivations below but, as they are rare, there is little impact if they are included in practice. For the derivation of our estimators, we also assume that at most one mutation occurs at each site since the MRCA of the sequences at that site. The effect of this assumption is minor when the mutation rate is low, which is the case for humans and many other organisms. We further assume that sequencing errors occur independently at rate ϵ at each site, and do not occur at non-ancestral alleles.

Suppose that a recombination occurs at site s of sequence c_1 creating two subsequences going backward in time, in the intervals $[1, s]$ and $[s{+}1, \ell]$. This recombination is effective for (c_1, c_2) if and only if one of these subsequences coalesces (reaches a common ancestor) with c_2 before coalescing with the other subsequence. By symmetry, all three possible coalescence events are equally likely, and so the recombination has probability 2/3 of being effective. Mutation and effective recombinations occur independently at each site of c_1 and c_2. Given that one of these events occurs at a site before the two sequences reach their MRCA, with probability $(2r/3)/(\mu{+}2r/3)$ it is an effective recombination. Thus the expected number of mutations that occur in the segment before it is terminated by an effective recombination is one less than the mean of a geometric distribution with parameter $(2r/3)/(\mu{+}2r/3)$, which is $3\mu/2r$. Site differences in IBDs can also arise from sequencing errors, which occur with rate 2ϵ per site.

Let $\bar{L} = \sum_{i=1}^{I}(r_i{-}l_i)/I$ and $\bar{M} = \sum_{i=1}^{I}|M_i|/I$ denote the averages of the IBD segment length and the number of sites that differ in an IBD segment, respectively. Our first estimating equation is

$$\bar{M} = \frac{3\hat{\mu}}{2r} + 2\bar{L}\hat{\epsilon}. \tag{1}$$

which can be read intuitively as site differences = mutations + sequencing errors. Our second estimating equation has a similar interpretation, but is based on site differences on each sequence relative to both its neighbours in the efficient subset, rather than between pairs of sequences:

$$C_1 = C_2\frac{\hat{\mu}}{r} + m\ell\hat{\epsilon}, \tag{2}$$

where we define

$$C_1 = \frac{3}{2}\sum_{\substack{i,j=1\\i<j}}^{I}\sum_{s=1}^{\ell}\Big\{\mathbb{I}(s\in M_i{\cap}M_j)\times\mathbb{I}(c_{i2}=c_{j1})\times\mathbb{I}(p_i\neq p_j)\Big\} \quad (3)$$

$$C_2 = \frac{9}{4}\sum_{\substack{i,j=1\\i<j}}^{I}\sum_{\substack{i',j'=1\\i'<j'}}^{I}\sum_{s=1}^{\ell}\Bigg\{\mathbb{I}(c_{i2}=\;c_{j1}=c_{i'2}=c_{j'1})\times\mathbb{I}(s={r_i}=r_j=l_{i'}=l_{j'})\times$$

$$\mathbb{I}(p_i\neq p_j\cup p_{i'}\neq p_{j'})\Bigg\}. \quad (4)$$

These quantities estimate, respectively, the total number of sequencing errors and mutations (C_1) and the number of recombinations (C_2) on the branch immediately above sequence c, before the first coalescence between any of $\{c{-}1, c, c{+}1\}$. See below for further explanation. Among all of the target recombinations, only 4/9 of them can be unambiguously determined from the data, so we scale this count by 9/4 in (4). The factor $\hat{\mu}/r$ in (2) converts the estimated number of recombinations to an estimated number of mutations. The final term in (2) is the expected total number of sequencing errors among the m sequences, each of length ℓ.

Combining (1) and (2), we obtain

$$\hat{\mu} = \frac{2m\ell\bar{M}-4\bar{L}C_1}{3m\ell-4\bar{L}C_2}r, \qquad \hat{\epsilon} = \frac{3C_1-2C_2\bar{M}}{3m\ell-4\bar{L}C_2}. \quad (5)$$

Present and past population sizes

We first estimate g_i, the TMRCA of c_{i1} and c_{i2}. Given g_i, the probability of a recombination event being effective (and thus being the right end point of the IBD) is no longer 2/3, but a function of g_i. For example, if a recombination event occurs more recently than the coalescence, when g_i is small there is little opportunity for the two subsequences created by the recombination to find a common ancestor before g_i, which implies a high probability for this recombination event to be effective. For this reason, it is difficult to estimate g_i based either on recombination events or the distribution of IBD lengths.

Note that $|M_i|$ follows a Poisson distribution with parameter $2(\mu g_i+\epsilon)(r_i-l_i)$. Given $\hat{\mu},\hat{\epsilon}$, we find the first moment estimator of g_i by solving, for $p\in P$,

$$\sum_{i=1}^{I}|M_i|\mathbb{I}(p_i=p) = (2\hat{\mu}g_p+2\hat{\epsilon})\sum_{i=1}^{I}(r_i-l_i)\mathbb{I}(p_i=p)$$

to obtain

$$\tilde{g}_p = \frac{1}{\hat{\mu}}\left(\frac{\sum_{i=1}^{I}|M_i|\mathbb{I}(p_i=p)}{2\sum_{i=1}^{I}(r_i{-}l_i)\mathbb{I}(p_i=p)}-\hat{\epsilon}\right).$$

Let $g_0=0$, and noting that $g_{p-1}<g_p$ for $p=m{+}2,\ldots,n$, we estimate g_p by the following quadratic optimization with linear constraints,

$$\check{g}_p = \underset{g_p}{\text{argmin}}\|g_p-\tilde{g}_p\|_2^2.$$

$$\text{subject to } g_{p'}-g_p\geq 0, \quad \text{for } p'>p \text{ and } p',p\in P.$$

By the nature of constrained optimization, many parent nodes will be inferred to share the same age, which is unrealistic. Numerical studies show that it will be helpful to

smooth these estimates when they are used later in estimating the population size. For this reason, the final estimate $\hat{g}_p$ of g_p is acquired by further smoothing $\check{g}_p$ by a Savitzky-Golay smoothing filter [1].

Note that this requires us to know the correct ordering of the (often incomparable) internal nodes of the TS. While this information is retained in the true TS, it is very difficult to infer from sequences, so direct inferences on TMRCA (and downstream inferences) are unreliable on inferred TS.

We estimate population sizes by first estimating the density $f_{\tilde{G}}$ of $\tilde{G}$, the TMRCA at a specific site s. We estimate $f_{\tilde{G}}$ by relating it to the density f_G of G, the TMRCA of an IBD segment. We first estimate f_G empirically from the estimated TMRCAs of each IBD segment. Then the density of $\tilde{G}$ is derived by conditioning on L, the length of the IBD segment:

$$f_{\tilde{G}}(g) = \frac{\sum_{l\geq 1} l f_{G|L}(g|L)\Pr(L=l)}{\mathrm{E}(L)} = \frac{\sum_{l\geq 1} l \Pr(L=l|G=g) f_G(g)}{\mathrm{E}(L)} = \frac{\mathrm{E}(L|G=g)}{\mathrm{E}(L)} f_G(g).$$

The mean IBD length $\mathrm{E}(L)$ can be estimated as $\bar{L}$, and the conditional mean $\mathrm{E}(L|G)$ can be found by a local linear kernel regression estimator given each pair $(\hat{g}_{p_i}, r_i - l_i)$ of IBD_i, where $\hat{g}_{p_i}$ is the estimated TMRCA. Thus, the estimate $\hat{f}_{\tilde{G}}$ of $f_{\tilde{G}}$ can be found by substituting the corresponding estimates of f_G, $\mathrm{E}(L|G)$ and $\mathrm{E}(L)$. We then smooth the estimate $\hat{f}_{\tilde{G}}$ by a Savitzky–Golay filter.

To estimate population sizes, note that the distribution of $\tilde{G}$ is solely determined by the coalescent rate $1/N(g)$, i.e.,

$$\Pr(\tilde{G} > g) = \exp\left\{-\int_0^g \frac{1}{N(t)}\mathrm{d}t\right\}.$$

Taking the log-derivative with respect to g on both sides, we have

$$N(g) = \frac{1 - F_{\tilde{G}}(g)}{f_{\tilde{G}}(g)}.$$

We thus calculate the estimate

$$\hat{N}(g) = \frac{\int_g^\infty \hat{f}_{\tilde{G}}(t)\mathrm{d}t}{\hat{f}_{\tilde{G}}(g)},$$

followed by another Savitzky–Golay smoothing filter.

Interpretation of C_1 and C_2

The quantity C_1 estimates the total number of sequencing errors and mutations on the branch immediately above (i.e. backwards in time from) a sequence c, before the first coalescence between any of c, $c-1$ and $c+1$.

In the efficient IBD subset, for each sequence c we record the IBDs of the pairs $(c-1, c)$ and $(c, c+1)$. If IBDs in $(c-1, c)$ and $(c, c+1)$ covering a site s have different parent nodes, then c must coalesce with either $c-1$ or $c+1$ at site s more recently than the coalescence of $c-1$ with $c+1$. In this case, any site differences contained in both IBDs can be attributed to an event unambiguously located on the branch immediately above sequence c before the first coalescence.

We also wish to include site changes above c in the case where $c-1$ coalesces with $c+1$ first, so that site differences are not available. By symmetry, this case occurs 1/3 of the time, and so we scale the previous count by a factor of 3/2. See S6 Fig (top left) for illustration. The quantity C_1 in (3) thus sums the total number of sequencing errors

and mutations on the branch immediately above each sequence c, before the first coalescence.

Likewise, the quantity C_2 estimates the total number of recombinations on the branch immediately above each sequence c, before the first coalescence between any of c, $c{-}1$ and $c{+}1$. Recall that p_i, l_i and r_i are the MRCA and left and right endpoints of IBD_i. Similarly to C_1, we only count recombinations that produce four adjacent IBDs IBD_i, $\mathrm{IBD}_{i'}$, IBD_j and $\mathrm{IBD}_{j'}$, with the first two corresponding to sequence pair $(c{-}1, c)$ and the latter two corresponding to $(c, c{+}1)$, such that $s = r_i = l_{i'} = r_j = r_{j'}$ (i.e., the breakpoint between i and i' is the same as the breakpoint between j and j'), and either $p_i{\neq}p_j$ or $p_{i'}{\neq}p_{j'}$, as shown in S6 Fig (top right). We then scale to account for the remaining cases.

If we have two IBD breakpoints at s, we must have a recombination on the c lineage since we assume that only one recombination can occur at s. If the MRCAs of these IBDs also satisfy $p_i{\neq}p_j$ or $p_{i'}{\neq}p_{j'}$, the recombination must occur before any coalescence, since:

- if $\{c{-}1, c{+}1\}$ coalesce before the recombination, we must observe $p_i = p_j$ and $p_{i'} = p_{j'}$;
- if $\{c{-}1, c\}$ coalesce before the recombination, there would not be an IBD breakpoint at s for the $(c{-}1, c)$ pair;
- likewise for when $\{c, c{+}1\}$ coalesce before the recombination.

Thus we do indeed count a subset of the desired recombinations.

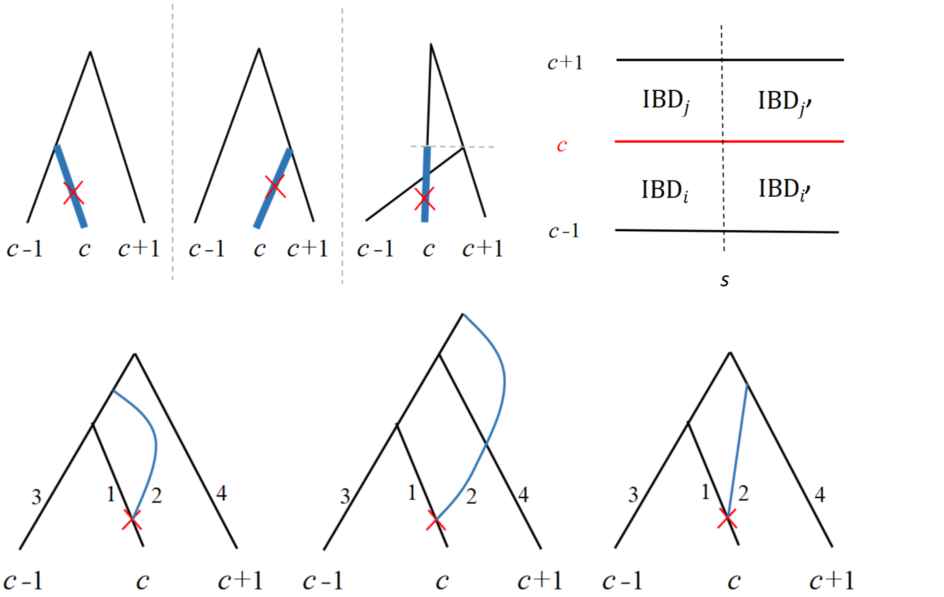

Fig S6. Top left: the three possible coalescent patterns of sequences c, $c{-}1$ and $c{+}1$ at a site s. While mutation events on the thicker edges should be included in the quantity C_1, only those in the first two patterns are counted. Top right: a sketch of four IBDs corresponding to sequence pairs $(c{-}1, c)$ and $(c, c{+}1)$. Bottom: a recombination event occurred on sequence c, which breaks the sequence before any coalescence between $c{-}1$, c and $c{+}1$, immediately resulting in a total of four segments $(1, 2, 3, 4)$. The figure shows three of the possible coalescent patterns, corresponding to the cases where segments 1 and 3 coalesce first.

When there is a recombination in c before any coalescences, there are 4 lineages immediately after the recombination (backwards in time), as shown in S6 Fig (bottom). There are three cases:

- If lineages 1 and 2 coalesce first, the recombination is not effective and there are no IBD breakpoints at s (probability 1/6).
- If lineages 3 and 4 coalesce first, we will have $p_i = p_{i'}$ and $p_j = p_{j'}$ and so not count the recombination (probability 1/6).
- Otherwise, we may count the recombination (probability 2/3).

As shown in S6 Fig (bottom), suppose (without loss of generality) that for the third case, lineages 1 and 3 coalesce first. If the coalesced lineage then coalesces with lineage 2, then there will not be an IBD breakpoint at s for $(c, c{+}1)$; otherwise the required pattern will be produced. Thus we only count $2/3 \times 2/3 = 4/9$ of the cases, and so scale by a factor of 9/4 to estimate the desired number of recombinations.

References

1. Savitzky A, Golay MJ. Smoothing and differentiation of data by simplified least squares procedures. Analytical Chemistry. 1964;36(8):1627–1639.
