# Supplementary material for: Estimating evolutionary and demographic parameters via ARG-derived IBD
Source: PLoS Genet. 2025 Jan 8;21(1):e1011537. doi: 10.1371/journal.pgen.1011537 (PMC11750106; doi:10.1371/journal.pgen.1011537)
Supplement: S3 Text — (PDF) [file pgen.1011537.s003.pdf]

Estimating evolutionary and demographic parameters via ARG-derived IBD

Text S3: Different models for sequencing error

In the main simulations we only allowed sequencing errors from 0 to 1. Errors from 1 to 0 are typically rare, because 1 alleles are rare and because there are four underlying nucleotides: if a site has mutated away from the ancestral nucleotide, a subsequent error may be to a different nucleotide rather than a return to the ancestral state. To verify that including 1 to 0 errors has little impact, we performed additional simulations. Results from Case C (Table **??**), which corresponds to the scenario in which errors from 1 to 0 arise in the observed data but not in the TSABC simulations, suggest some underestimation of μ as expected; the effect is small and in this study non-significant. Case A corresponds to the simulation studies included in the paper, while Case B allows 1 to 0 errors both in the dataset treated as observed and the TSABC simulation model. Case A and Case B results are virtually indistinguishable, both show good estimation of both μ and ϵ, with Case C showing slightly worse precision.

	Case A	Case B	Case C
$\hat{\mu} \times 10^8$ (SE)	1.313 (0.025)	1.324 (0.024)	1.272 (0.037)
$\hat{\epsilon} \times 10^4$ (SE)	0.987 (0.037)	0.984 (0.025)	1.029 (0.049)

Table S1. Case A: both the dataset treated as observed and the datasets simulated within TSABC do not include errors from 1 to 0. Case B: both the observed and the TSABC datasets include errors from 1 to 0 (at the same rate). Case C: the observed dataset includes errors from 1 to 0 while the TSABC datasets do not. Each sequencing error swaps an ‘A’, ‘T’, ‘C’ or ‘G’ to one of the other three nucleotides chosen with equal probability. Here $\mu = 1.3 \times 10^{-8}$, $\epsilon = 10^{-4}$, $\ell = 10^6$, $n = 10$, $r = 10^{-8}$ and $N(g) = 20\,000$ for all $g \geq 0$ (Model C). Results are based on TSABC analysis of 25 observed datasets for each case.
