# Supplementary material for: Estimating evolutionary and demographic parameters via ARG-derived IBD
Source: PLoS Genet. 2025 Jan 8;21(1):e1011537. doi: 10.1371/journal.pgen.1011537 (PMC11750106; doi:10.1371/journal.pgen.1011537)
Supplement: S4 Text — (PDF) [file pgen.1011537.s004.pdf]

Estimating evolutionary and demographic parameters via ARG-derived IBD

Text S4: Further details for 1KGP data analysis

The chromosome lengths are $\ell_{20} = 63\,025\,522$ and $\ell_{21} = 48\,129\,897$ sites, of which 1 552 394 and 927 753 sites are polymorphic in the full dataset. The sequence data were downloaded as .vcf files from ftp.1000genomes.ebi.ac.uk. Then, we converted them to the .samples format required for input to tsinfer and adopted human reference assembly GRCh37 recombination map following the data pre-processing steps in [1]. Specifically, we first cloned the Github repository from github.com/awohns/unified_genealogy_paper and installed all of the necessary software, packages and modules listed in the "requirements.txt" file and the "tools" sub-folder. Then we redirected to the "all-data" sub-folder and conducted the "Makefile" document to build the tree sequence for 1000 Genomes chromosome 20, during which the program downloaded the chromosome 20 variant data and produced a .samples file (tsinfer input format) converted from a .vcf file. Then IBDs were extracted from the inferred TS and supplied to TSABC. The process was repeated for chromosome 21.

References

1. Wohns AW, Wong Y, Jeffery B, Akbari A, Mallick S, Pinhasi R, et al. A unified genealogy of modern and ancient genomes. Science. 2022;375(6583):eabi8264.
