# Supplementary figures and images for: Estimating evolutionary and demographic parameters via ARG-derived IBD
Source: PLoS Genet. 2025 Jan 8;21(1):e1011537. doi: 10.1371/journal.pgen.1011537 (PMC11750106; doi:10.1371/journal.pgen.1011537)

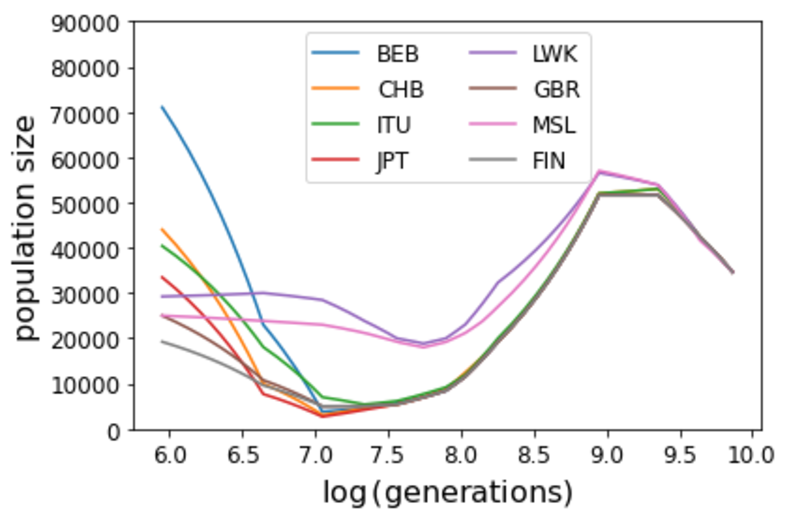

Supplement: S1 Fig — Natural logarithms of g are shown on the x-axis, with the models starting at g = exp(6) ≈ 400 generations in the past. The values of N(1000) which form the right endpoints of Fig 7 correspond to x = log(1000) ≈ 6.9. (TIF) [file pgen.1011537.s005.tif]

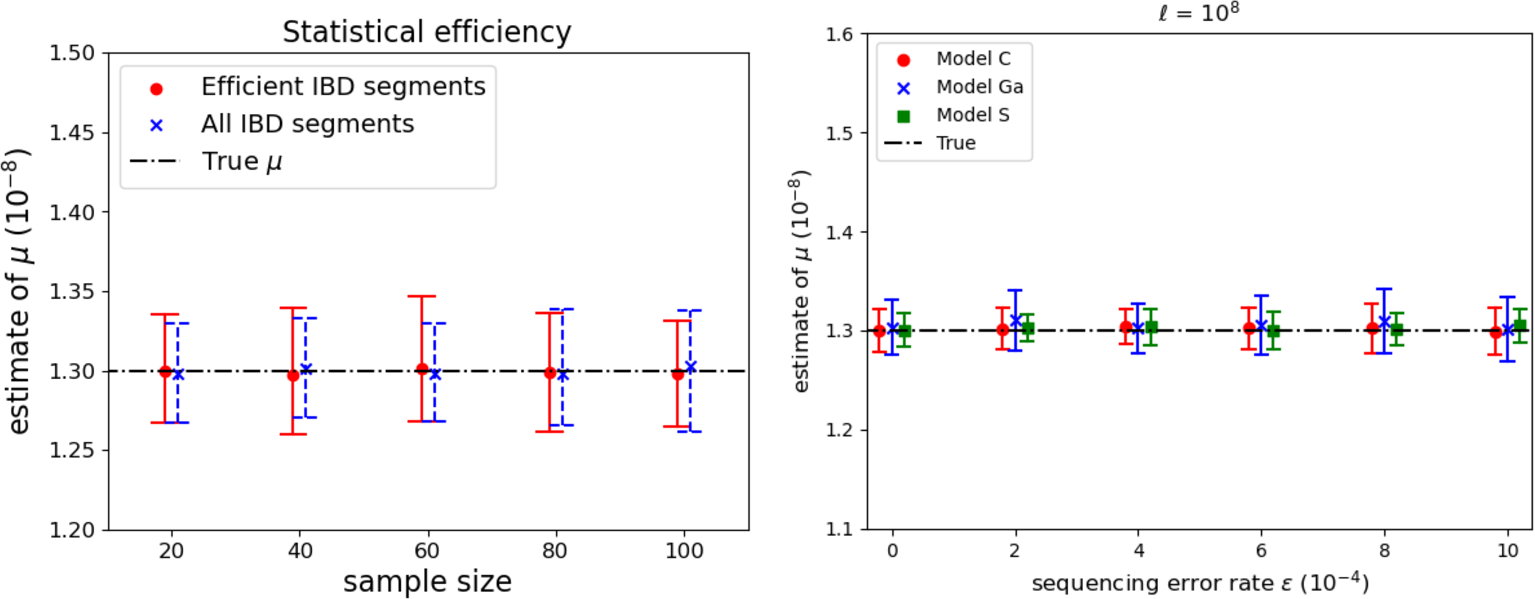

Supplement: S2 Fig — Left: Estimated 95% CIs for the estimation of μ when an efficient subset of IBD segments was extracted from the TS and when all IBDs were used. At each sample size, 25 replicate datasets were simulated under Model C, with sequence length ℓ = 107. Right: Impact of sequencing error rate ϵ on μ^ when ℓ = 108 (other details are the same as for bottom left panel of Fig 3). (TIF) [file pgen.1011537.s006.tif]

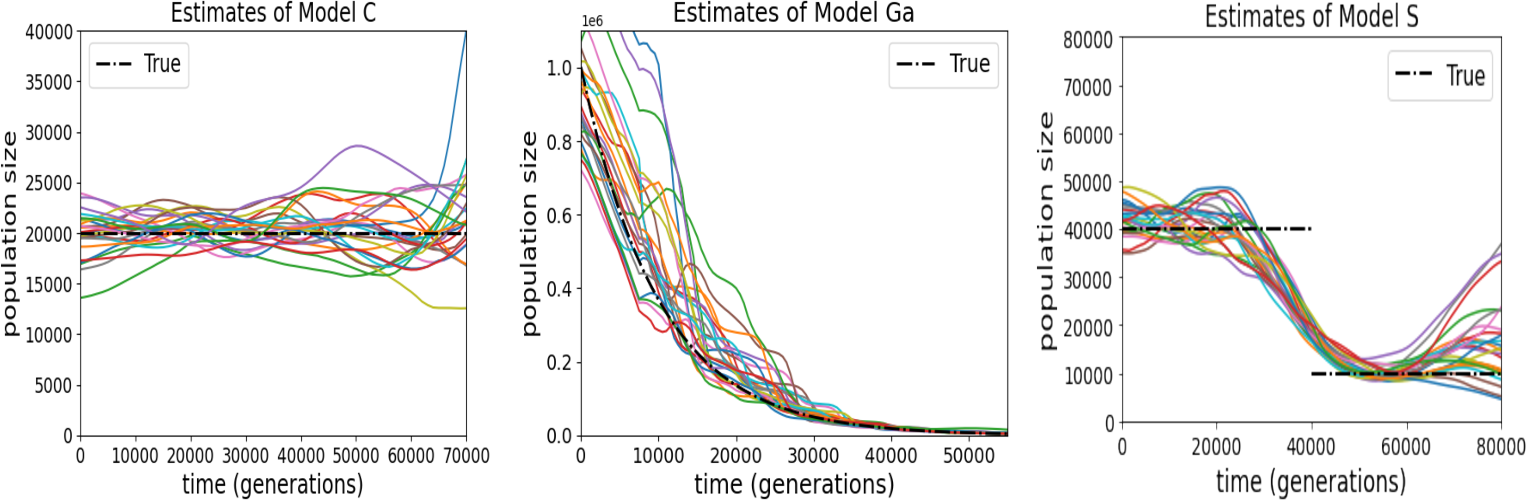

Supplement: S3 Fig — (TIF) [file pgen.1011537.s007.tif]

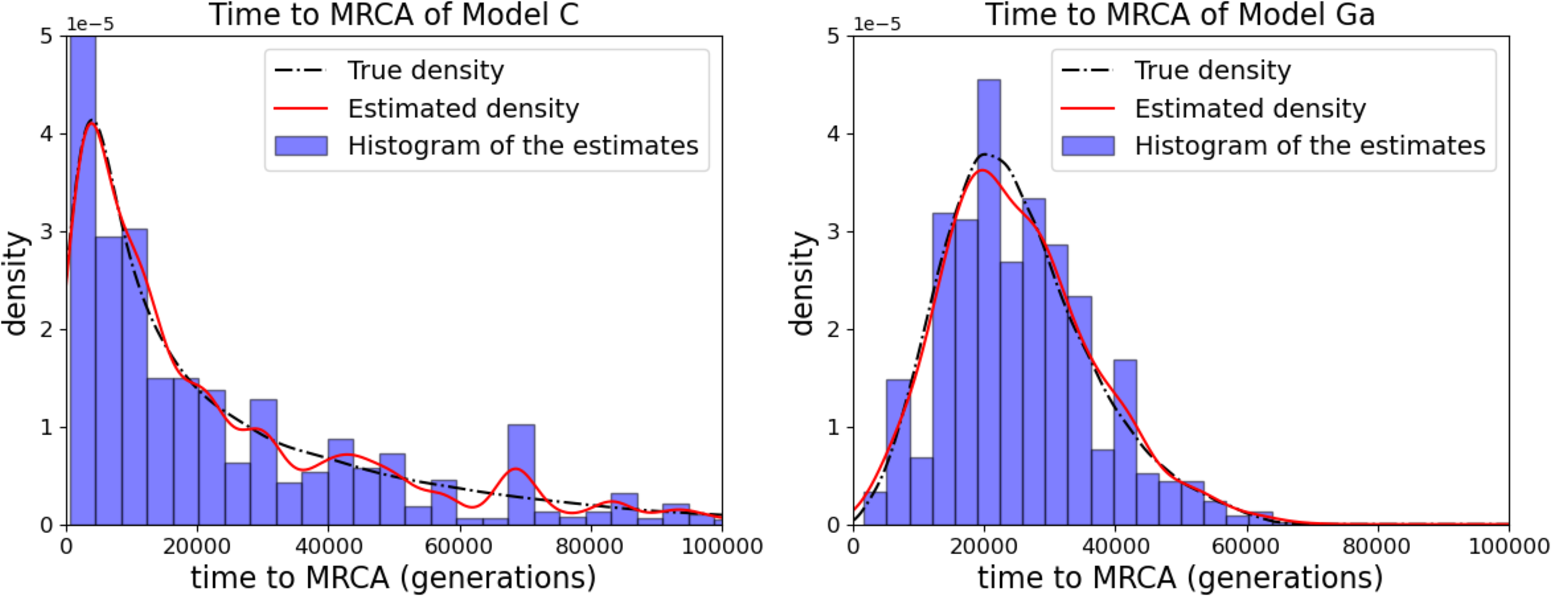

Supplement: S4 Fig — Also shown is a probability density obtained by kernel smoothing of the gi^ together with the true density. True IBD was available for inference but no time information. (TIF) [file pgen.1011537.s008.tif]

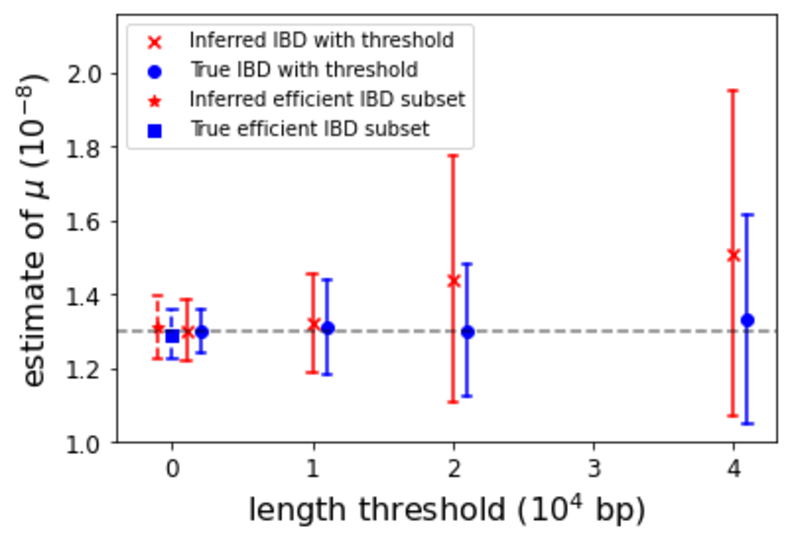

Supplement: S5 Fig — (TIF) [file pgen.1011537.s009.tif]
